# Community analysis of the abundance and diversity of biting midge species (Diptera: Ceratopogonidae) in three European countries at different latitudes

**DOI:** 10.1186/s13071-018-2792-x

**Published:** 2018-03-27

**Authors:** Tim W. R. Möhlmann, Uno Wennergren, Malin Tälle, Guido Favia, Claudia Damiani, Luca Bracchetti, Willem Takken, Constantianus J. M. Koenraadt

**Affiliations:** 1 Laboratory of Entomology, Wageningen University and Research, P.O. Box 16, 6700 AA Wageningen, the Netherlands; 2 IFM Theory and Modelling, Linköping University, 581 83 Linköping, Sweden; 3 Scuola di Bioscienze e Medicina Veterinaria, Università degli Studi di Camerino, 62032 Camerino, Italy

**Keywords:** *Culicoides*, Midge sampling, Species diversity, OVI trap, Community ecology

## Abstract

**Background:**

The outbreaks of bluetongue and Schmallenberg disease in Europe have increased efforts to understand the ecology of *Culicoides* biting midges and their role in pathogen transmission. However, most studies have focused on a specific habitat, region, or country. To facilitate wider comparisons, and to obtain a better understanding of the spread of disease through Europe, the present study focused on monitoring biting midge species diversity in three different habitat types and three countries across Europe.

**Methods:**

Biting midges were trapped using Onderstepoort Veterinary Institute light traps at a total of 27 locations in Sweden, the Netherlands and Italy, comprising farm, peri-urban and wetland habitats. From July 2014 to June 2015 all locations were sampled monthly, except for during the winter months. Trapped midges were counted and identified morphologically. Indices on species richness, evenness and diversity were calculated. Community compositions were analysed using non-metric multidimensional scaling (NMDS) techniques.

**Results:**

A total of 50,085 female midges were trapped during 442 collection nights. More than 88% of these belonged to the Obsoletus group. The highest midge diversity was found in Sweden, while species richness was highest in the Netherlands, and most specimens were trapped in Italy. For habitats within countries, diversity of the trapped midges was lowest for farms in all countries. Differences in biting midge species communities were more distinct across the three countries than the three habitat types.

**Conclusions:**

A core midge community could be identified, in which the Obsoletus group was the most abundant. Variations in vector communities across countries imply different patterns of disease spread throughout Europe. How specific species and their associated communities affect disease risk is still unclear. Our results emphasize the importance of midge diversity data at community level, how this differs across large geographic range within Europe, and its implications on assessing risks of midge-borne disease outbreaks.

**Electronic supplementary material:**

The online version of this article (10.1186/s13071-018-2792-x) contains supplementary material, which is available to authorized users.

## Background

Worldwide around 1400 species of *Culicoides* (biting midges, Diptera: Ceratopogonidae) have been described [1]. At present, the European Interactive *Culicoides* Key [2, 3] includes 110 species. A minority of these species has thus far been described as important vectors for arthropod-borne viruses (arboviruses) [4, 5]. Biting midges from the *Culicoides* Obsoletus group [6–11], *C. imicola* Kieffer, 1913 [12], *C. pulicaris* (Linnaeus, 1758) [11, 13, 14], and *C. punctatus* (Meigen, 1804) [10, 14–19] are important vectors occurring in Europe. Of the over 50 viruses isolated from biting midges, several are of major international significance [4]. Animal diseases caused by viruses such as Akabane virus, bovine ephemeral fever virus, African horse sickness virus (AHSV) and bluetongue virus (BTV) are all transmitted by biting midges. Both infection with AHSV and bluetongue are of such international significance that they are listed by the World Organisation for Animal Health (OIE) as focus diseases that can have serious socio-economic or public health consequences, and are of major importance for international trade [20].

Outbreaks of midge-borne viruses in Europe, e.g. BTV since 2006, and more recently Schmallenberg virus (SBV) during 2011–2013 [21, 22], have had a great impact on the European livestock sector [23–25]. Milk production by infected livestock is often reduced, and the virus affects unborn calves and lambs when the mother becomes infected during gestation, resulting in non-viable offspring. These impacts, in addition to livestock movement restrictions, result in considerable economic losses. Factors such as intensified transportation of livestock and the rise in global temperature may further increase the risk of arbovirus outbreaks in Europe. Rising temperatures create opportunities for vector populations to increase rapidly, and allow viruses to complete their extrinsic incubation period in vectors faster, which both imply an increased potential for pathogen transmission [26].

Although research following the outbreaks of BTV and SBV has improved our knowledge of the ecology of *Culicoides* biting midges, these studies often focus on a specific habitat, region, or country [27–31]. Therefore, it is difficult to make direct comparisons among the results of these studies. Thus, to facilitate wider comparisons on biting midge communities at European level, we aimed to simultaneously sample midge species distribution, abundance, and diversity in three habitat types within three representative countries at different latitudes in Europe, where large differences in environmental characteristics could be expected.

## Methods

### Midge trapping

Adult midges were trapped using Onderstepoort Veterinary Institute (OVI) light traps. A 30 cm 8 W fluorescent black light tube was used to attract midges [1]. When in close proximity of the trap, midges were sucked in by the down-draught fan, which was powered by the main grid or a 12 V, 24–32 Ah battery [14, 17, 32]. The top of the trap was placed at a height of 1.5–2 m and traps were at least 100 m apart to prevent interference between them. The collection bucket had a capacity of 500 ml and larger insects were excluded by polyester netting (mesh size 2–4 mm) placed around the light source of the trap. The bucket was filled with 50 ml water-soap solution.

### Sampling procedures

Traps were placed in three countries at different latitudes: Sweden (surroundings of Linköping 58°24'38.9"N, 15°37'17.5"E), the Netherlands (surroundings of Wageningen 51°57'53.3"N, 5°39'46.4"E), and Italy (surroundings of San Benedetto del Tronto 42°56'58.1"N, 13°52'42.6"E). Within each country, farm, peri-urban and wetland habitats were selected. Selection criteria for habitat type and trap location have been described in [33]. In brief, each habitat type was represented by three unique sampling locations. Traps were positioned in these locations within 50 m of open stables of dairy cattle (‘farm’), a residential property (‘peri-urban’) or standing water (‘wetlands’). Habitat types mostly matched the classification of the CORINE European Land cover database [34].

From July 2014 to June 2015, except for the months December, January and February (and March for Sweden), monthly collections were performed for six consecutive days in each of the countries. Traps were active for 24 h and were emptied and rotated among the sampling locations between 08:00 h and 17:00 h the next day. Midges were sorted and stored at -20 °C in Eppendorf tubes containing 70% ethanol solution.

### Sample identification

All female midges were identified to species level in collections that contained less than 100 individual midges. For collections that contained more than 100 midges (14% of the collections), a random sub-sample of at least 50 individuals was identified as an estimation of the species composition of the total collection. All identifications were performed using the Interactive Identification Key for *Culicoides* (IIKC) [2, 3]. Morphologically similar species were recorded as belonging to a group or complex [35, 36].

### Statistical analyses

Species diversity indices were calculated with the Simpson’s Index of Diversity: $1-D=1-\frac{\sum {n}_i\left({n}_i-1\right)}{N\left(N-1\right)}$, Shannon-Wiener Diversity Index: ${H}^{\prime }=-\sum \limits_{i=1}^R{p}_i\ln \left({p}_i\right)$, and the Shannon-Wiener evenness: $E=\frac{H^{\prime }}{\ln (S)}$ . Diversity indices were calculated for each of the three countries as well as for farm, peri-urban and wetland habitats. To better understand whether sufficient trapping efforts had been made for a reasonable estimate of species diversity, a rarefaction curve of the species and the number of trapped midges was created with the rarecurve function within the *vegan* version 2.9.2. [37] package in R version 3.2.3. [38].

Non-metric multidimensional scaling (NMDS) analyses were used to evaluate the combined effects of country, habitat and diversity on the midge community composition [39]. An NMDS analysis can deal with abundant null measures in a dataset and calculates a reliable best model fit for shortest distances between the elements. The degree of stress calculated within this analysis indicates the reliability of the plot that is generated with NMDS, whereby lower stress corresponds to a higher reliability of the plot. For values above 0.3 the NMDS ordination plot is considered arbitrary. For NMDS analyses, the metaMDS function with the Bray-Curtis dissimilarity metric was used. All data were analysed using the statistical software package R version 3.2.3. [38].

## Results

A total of 442 trap collections were performed in Sweden, the Netherlands and Italy (Table 1). In 305 (69%) of these collections one or more biting midges were trapped, whereas in the remainder of the collections (31%), no biting midges were trapped. A total of 50,729 biting midges were trapped during this study. Of these specimens, 7818 (15.4%) female midges were identified to species level. After identification, a total of 50,085 female (98.7%) biting midges were estimated to be trapped. Other individuals either were males or damaged to the extent that they could not be identified morphologically. A total of 45 midge species were found for the three countries combined. Of all female biting midges trapped during the field study, the number of specimens trapped was highest for the Obsoletus group (88.6%), followed by *C. punctatus* (2.3%), *C. pulicaris* (2.2%) and *C. festivipennis* (Kieffer 1914) (1.6%).

**Table 1:** Midge species diversity. Estimators of taxonomic diversity with values for the Simpson’s Index of Diversity, Shannon-Wiener diversity and Shannon-Wiener evenness for three habitats (farms, peri-urban and wetlands) in three countries (Sweden, the Netherlands and Italy)

Taxonomic diversity	Sweden				the Netherlands				Italy				Total
	Farm	Peri-urban	Wetland	Total	Farm	Peri-urban	Wetland	Total	Farm	Peri-urban	Wetland	Total	
No. of specimens trapped	3267	46	761	4074	8270	185	3530	11,985	33,682	124	220	34,026	50,085
No. of samples	42	48	46	136	52	53	50	155	47	51	53	151	442
No. of species trapped	15	12	14	18	18	14	29	35	12	11	14	20	45
Simpson Index of Diversity	0.640	0.821	0.786	0.723	0.439	0.828	0.777	0.596	0.036	0.715	0.748	0.051	0.285
Shannon-Wiener diversity	1.439	2.000	1.801	1.714	0.807	2.051	1.863	1.386	0.127	1.589	1.750	0.178	0.813
Shannon-Wiener evenness	0.531	0.805	0.682	0.593	0.279	0.777	0.553	0.390	0.051	0.663	0.663	0.060	0.214

**Fig. 1:**
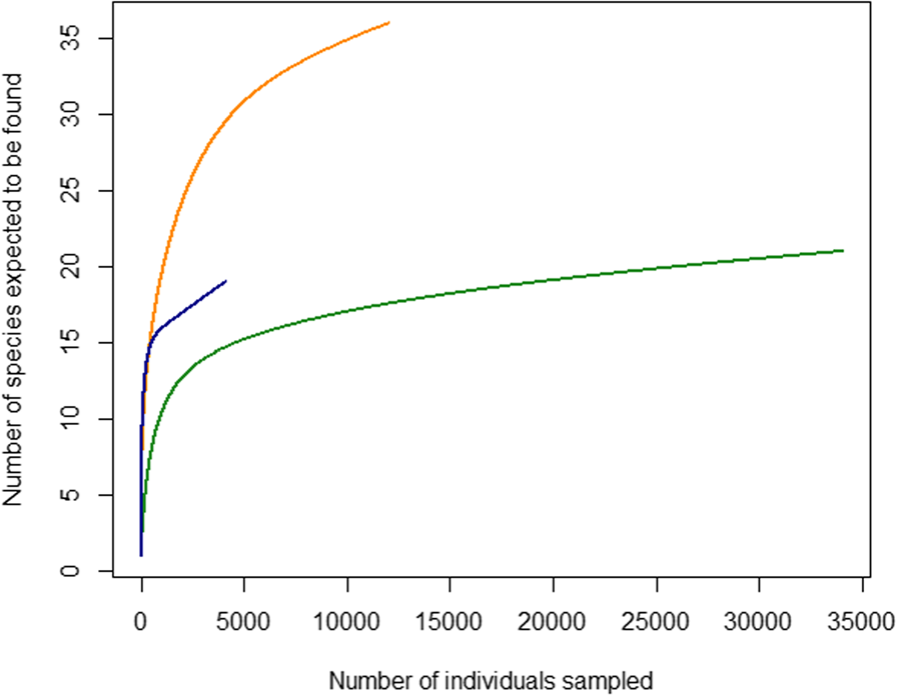
Rarefaction plot of sampling effort. The plot shows the number of species expected to be found for the number of individuals sampled for Sweden (blue), Italy (green) and the Netherlands (orange)

The rarefaction curves for each of the three countries are beyond their exponential growth and start to level off (Fig. 1). Although more sampling efforts would increase the number of species expected to be found (mostly in Sweden and the Netherlands), we believe that our sampling effort was sufficient for obtaining a representative number of species for the three countries.

Although the lowest numbers of samples and specimens were trapped in Sweden, the highest species diversity was found there. The lowest values of species diversity were found in Italy, but most specimens were trapped in this country (Table 1). The lowest midge species diversity was found at farms in all countries. The highest midge species diversity was found for midges trapped in peri-urban habitats in Sweden and the Netherlands, while the midge diversity was almost similar for peri-urban and wetlands in Italy (Table 1). Catches from peri-urban habitats had the lowest number of specimens, while the highest number of specimens were trapped at farms in all countries.

From the 4074 female midges trapped in Sweden, 18 species were identified. The most common species were from the Obsoletus group (47%), *C. pulicaris* (22%) and *C. achrayi* (Kettle & Lawson, 1955) (10%) (Table 2). The dominating species among the 3,267 females trapped on farms were from the Obsoletus group (54%) and *C. pulicaris* (27%). From the 46 female midges trapped in peri-urban habitats, *C. kibunensis* (Tokunaga, 1937) (28%) and *C. vexans* (Staeger, 1839) (22%) were the most common, whereas the 761 specimens from wetlands in Sweden were dominated by *C. achrayi* (31%) and *C. festivipennis* (27%).

**Table 2:** Midge species abundance. List of midge species with number of specimens for each country (Sweden, the Netherlands and Italy) and habitat type (farms, peri-urban and wetlands)

Species list	Sweden				the Netherlands				Italy				Total
	Farm	Peri-urban	Wetland	Total	Farm	Peri-urban	Wetland	Total	Farm	Peri-urban	Wetland	Total	
*C. achrayi*	173	1	235	409	7	20	23	50	41	2	1	44	503
*C. alazanicus*							283	283		2		2	285
*C. albihalteratus*		1		1									1
*C. brunnicans*					4	30		34					34
*C. cameroni*	4	1	8	13	1		6	7			5	5	25
*C. caucoliberensis*							1	1		1		1	2
*C. chiopterus*	34	1		35	2001	15	119	2135					2170
*C. circumscriptus*			1	1	5		3	8	45	7	96	148	157
*C. derisor*							7	7					7
*C. dewulfi*					4			4					4
*C. duddingstoni*									116	34	18	168	168
*C. fagineus*	40		30	70									70
*C. fasciipennis*					3		81	84					84
*C. festivipennis*	42	1	207	250	10	10	524	544	2	4	1	7	801
*C. flavipulicaris*									54			54	54
*C. griseidorsum*									6	15	5	26	26
*C. grisescens*					163		8	171					171
*C. heliophilus*							1	1					1
*C. heteroclitus*									85			85	85
*C. impunctatus*	14		1	15		1		1					16
*C. indistinctus*							8	8					8
*C. jurensis*							1	1					1
*C. kibunensis*	64	13	67	144	2	58	207	267					411
*C. longipennis*							2	2			1	1	3
*C. lupicaris*	86	3	8	97	1			1	37			37	135
*C. manchuriensis*						2	7	9					9
*C. maritimus*										2	21	23	23
*C. newsteadi*					1	1	4	6					6
*C. nubeculosus*							6	6					6
Obsoletus group	1726	9	136	1871	5860	28	1329	7217	33,069	54	25	33,148	42,236
*C. pictipennis*		2	20	22		3	78	81					103
*C. picturatus*							5	5					5
*C. poperinghensis*	21		4	25	4		4	8					33
*C. pseudopallidus*											1	1	1
*C. pulicaris*	892	1	6	899	11		18	29	184	1		185	1113
*C. punctatus*	120	3	36	159	191	7	776	974	38	2	1	41	1174
*C. reconditus*						1	1	2					2
*C. remmi*											1	1	1
*C. riethi*	1			1	2		3	5					6
*C. salinarius*	16			16			5	5					21
*C. simulator*							18	18					18
*C. subfasciipennis*						5		5					5
*C. submaritimus*											39	39	39
*C. tauricus*							1	1					1
*C. vexans*	34	10	2	46		4	1	5	5		5	10	61

The Netherlands had the highest species richness with 35 species identified among 11,985 female midges trapped during the study period. The most common species were from the Obsoletus group (78%), *C. punctatus* (8%) and *C. festivipennis* (5%). For wetland and farm habitats the most abundant species were comprised of those species. The 185 midges trapped from peri-urban habitats were dominated by *C. kibunensis* (31%) and *C. brunnicans* (Edwards, 1939) (16%).

Of the 20 species trapped in Italy, the Obsoletus group was by far the most dominant with 97% of all 34,026 trapped female midges. The trapped midges from farm (33,682) and peri-urban (124) habitats were dominated by the Obsoletus group, although *C. pulicaris* (1%) and *C. duddingstoni* Kettle & Lawson, 1955 (27%) were also trapped more than other species in the two habitats, respectively. From the 220 midges trapped in Italian wetland habitats, *C. circumscriptus* Kieffer, 1918 (44%) and *C. submaritimus* Dzhafarov, 1962 (18%) were most abundant.

**Fig. 2:**
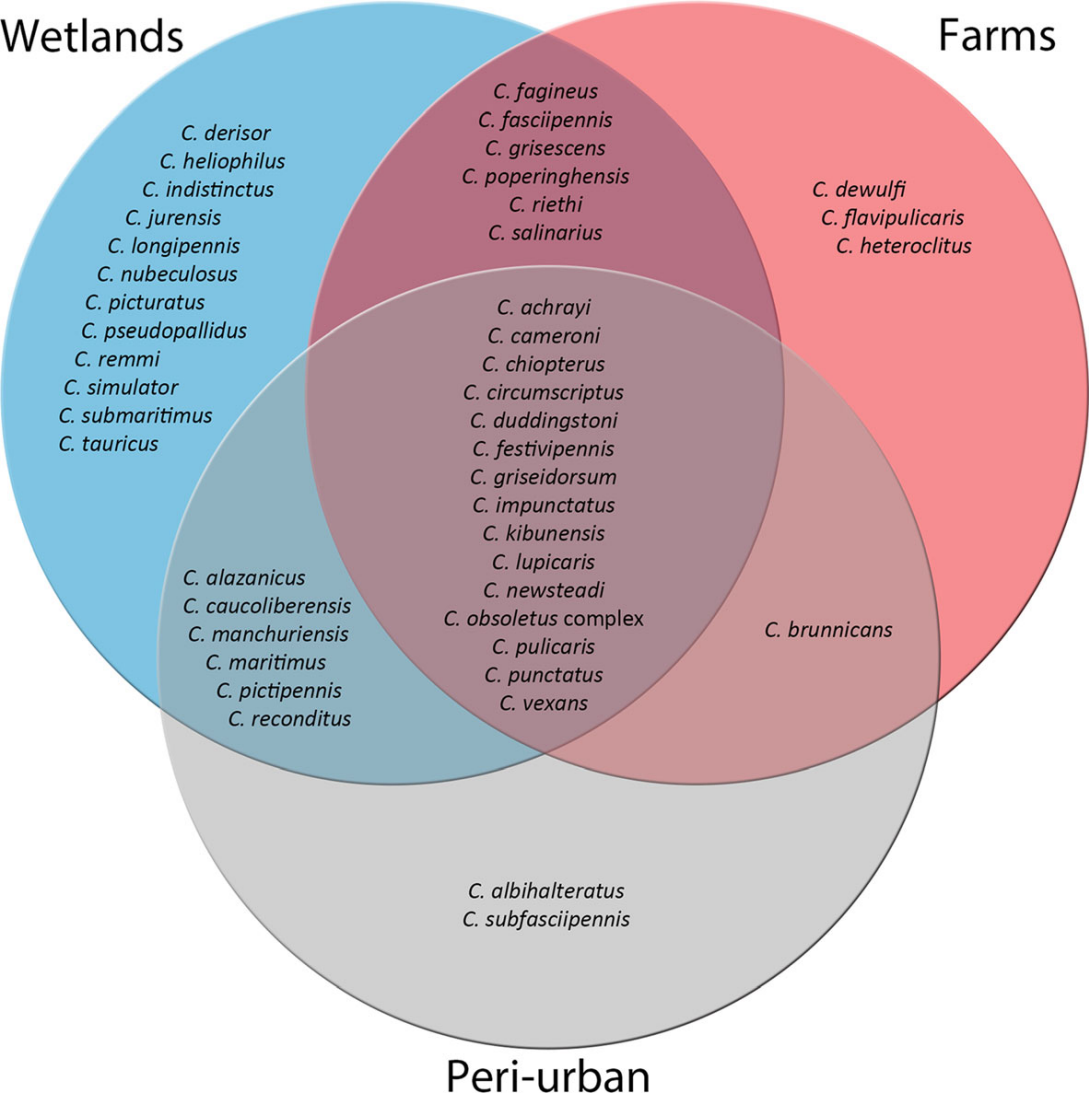
Venn diagram of habitats. Diagram shows the absolute presence of midge species found in farm (red), peri-urban (grey) and wetland (blue) habitats

**Fig. 3:**
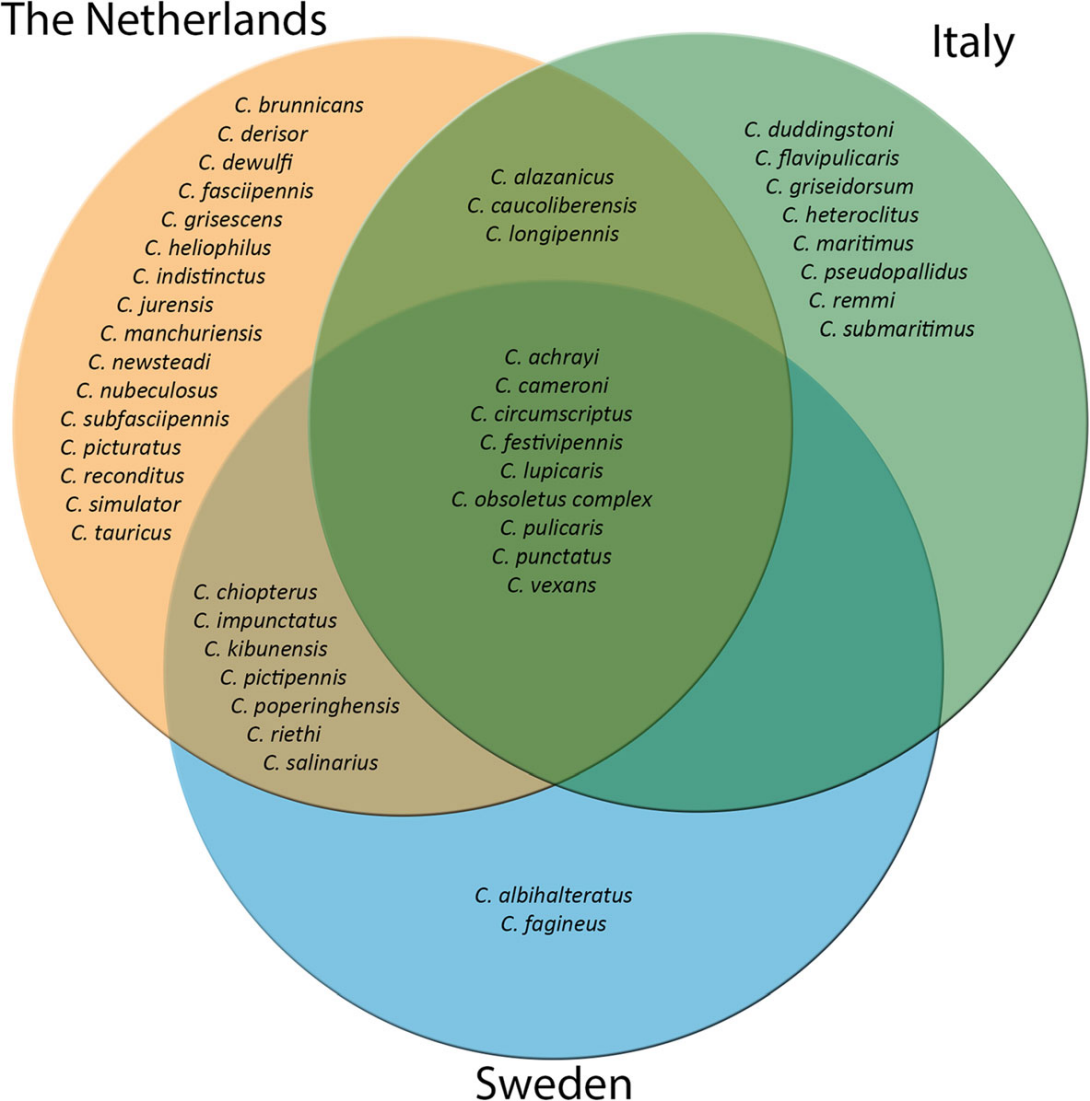
Venn diagram of countries. Diagram shows the absolute presence of midge species found in Sweden (blue), the Netherlands (orange) and Italy (green)

Of all midge species trapped, 38% (17/45) were unique to one of the three habitat types. Three species occurred exclusively at farms, two species in peri-urban habitats and 12 species only in wetland habitats (Fig. 2). Figure 3 shows that more than half of the 45 species identified were trapped in only one of the countries (26/45, 58%), while 20% (9/45) of the species were trapped in all three countries. This core community included the most abundant species from the three countries: the Obsoletus group, *C. punctatus*, *C. pulicaris*, *C. festivipennis* and *C. achrayi.*

**Fig. 4:**
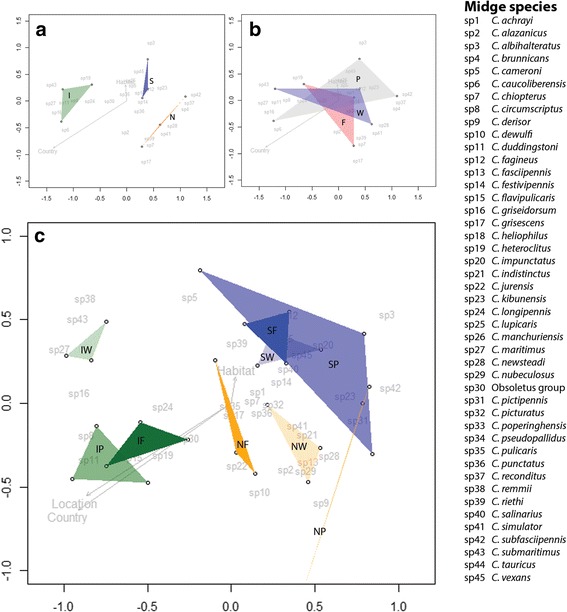
Results of NMDS analyses. **a** Figure shows midge community compositions for Sweden (S), the Netherlands (N) and Italy (I). **b** NMDS analysis for farms (F), peri-urban (P) and, wetland (W) habitats based on number of midges trapped per species in each habitat and country. **c** NMDS analysis based on number of midges trapped per species at each location in each country and habitat (Sweden in blue: SF, SP and SW; the Netherlands in orange: NF, NP and NW; Italy in green: IF, IP and IW). The Bray-Curtis dissimilarity index was used to determine dissimilarities among midge community compositions. Stress value = 0.084 for panels **a** and **b**, which indicates a very good fit of the model. Stress value = 0.216 for panel **c**, which indicates a suspect fit of the model

Combining the presence and abundance of different midge species trapped for the different countries and habitat types into one statistical analysis provides additional information about vector communities associated with specific regions. Dissimilarity matrices resulting from NMDS analyses show clear differences in biting midge community composition among the areas investigated in countries at different latitudes (*P* = 0.002, stress = 0.084) (Fig. 4a). Differences in communities were not found among habitats (*P* = 0.976, stress = 0.084) (Fig. 4b). However, looking at habitats within each country, midge communities were found to be different for some of the habitats (Fig. 4c). Midge communities among Dutch (*P* = 0.048, stress = 0.081) and Italian (*P* = 0.040, stress = 0.0594) habitats were significantly different from each other. For habitats in Italy this difference was mainly driven by the wetland habitat (Fig. 4c). Midge communities among habitats in Sweden were comparable (*P* = 0.577, stress = 0.099).

## Discussion

Biting midge community composition clearly differed among the areas we investigated at different latitudes. This is illustrated by the diversity indices (Table 1), which was highest in Sweden, followed by the Netherlands, and then Italy. In addition, the Venn-diagram (Fig. 3) shows that 57% of the trapped midge species were found in only one of the countries. Finally, the dissimilarity matrix (Fig. 4a) distinguishes distinct midge communities among countries. Although communities varied among the areas investigated for the countries, a core community of midges seems to be present nevertheless (Fig. 3). This core community includes the five most abundant species from the three countries: the Obsoletus group, *C. punctatus*, *C. pulicaris*, *C. festivipennis* and *C. achrayi.* While this core community occurs throughout Europe and across different habitats, it cannot be assumed that their contribution to disease spread is similar in all countries as temperature, interaction with other (host) species, and genetic variation within midge species [4, 40] also vary throughout Europe. However, these known midge vector species are present in a core community throughout Europe, and with rising temperature as a consequence of climate change, and continued increase in animal transport, it is expected that disease outbreaks will increase throughout Europe. Increasing temperature will simultaneously affect the rapid increase of midge vector populations, and at the same time allow viruses to complete their extrinsic incubation period in vectors faster, which both imply an increased potential for pathogen transmission [26].

While community composition clearly differed among the sampled areas at different latitudes, communities were similar among habitat types (Fig. 4a, b). However, when differentiating habitats within countries, there were marked habitat effects on community composition (Fig. 4c). Habitat communities from farm, wetland, and peri-urban sites differed within the Netherlands and Italy, while communities in Sweden were more similar to each other. These results are comparable to diversity and community composition found for mosquitoes in Europe [33]. Both mosquito and biting midge communities show clear differences among areas in the three representative countries, while these communities are different for habitats only within the countries studied. This suggests that local habitat factors can be important for vector community composition, but that ecological factors at large geographical distances between sites have a more significant impact.

Some of the biting midge species that were morphologically identified, were thus far not known to be present in the studied countries. Of 18 midge species identified for Sweden, two species (*C. cameroni* and *C. fagineus*) could not be confirmed by the IIKC [2] or literature [17, 41] (see Additional file 1: Table S1 for an overview). For the Netherlands, 12 species (*C. brunnicans*, *C. cameroni*, *C. caucoliberensis*, *C. derisor*, *C. indistinctus*, *C. jurensis*, *C. longipennis*, *C. manchuriensis*, *C. picturatus*, *C. reconditus*, *C. simulator* and *C. tauricus*) were not earlier described [10, 42, 43], and for Italy three species (*C. achrayi*, *C. cameroni* and *C. vexans*) were not found in literature [44, 45] or distribution maps of the IIKC. Because most of these species are known to be present in countries surrounding the countries studied here, we expect that the distribution is correct but was simply not confirmed before. We will continue to work with these samples and confirm the findings with barcoding techniques before adding them to current distribution lists.

From the European core midge community identified in this study, at least three species are (potential) vectors of pathogens. The Obsoletus group was the most abundant trapped in all countries, especially in farm habitats. Species in this group are known to transmit both BTV and SBV [6–11], and considered the most important midge vector species in Europe. However, the Obsoletus group consists of several species [*C. chiopterus* (Meigen, 1830); *C. dewulfi* Goetghebuer, 1936; *C. obsoletus* (Meigen, 1818) (*s.s.*); *C. scoticus* Downes & Kettle, 1952; and *C. montanus* Shakirzjanova, 1962] for which morphological identification is difficult and very laborious [41]. *Culicoides* identification remains a challenge, especially for specific groups or complexes of species [3, 36]. As new techniques such as DNA (barcode) sequencing and MALDI TOF (matrix assisted laser desorption/ionisation time of flight) mass spectrometry [31, 36, 46, 47] become available for identification, it will be easier to process large numbers of specimens. However, correct reference databases, morphological identifications, and the link with ecology remain essential components in *Culicoides* research. Therefore, the European Interactive Identification Key for *Culicoides* (IKCC) developed by Mathieu et al. [2] is a useful tool to obtain accurate morphological identifications. Those species that are difficult or impossible to separate by morphological identification, such as the Obsoletus group, can be further identified with molecular tools. Although species in the Obsoletus group are recognized as important potential vectors [48], it remains unknown what the species-specific (within the Obsoletus group) contribution to pathogen transmission is. Priority should therefore be given to investigate the Obsoletus group composition in more detail, to better understand disease dynamics.

The other two species found in this study that are (potential) vectors of pathogens were *C. punctatus* and *C. pulicaris.* These species were trapped in similar numbers and are known to transmit BTV and SBV [10, 11, 13–19]. Both species were found in all habitats and countries, although *C. punctatus* was mainly found in Dutch wetlands, while *C. pulicaris* was mostly present at farms in Sweden and Italy. One of the most important European BTV vectors for southern Europe, *C. imicola*, was not trapped during this study. Our trapping sites in Italy were further north compared to the known distribution of *C. imicola* [49], and results can therefore not be extrapolated for the most southern parts of Europe where *C. imicola* is present.

Although our sampling effort was comprehensive, as can be deduced from the rarefaction plot (Fig. 1), the study was carried out in a relatively limited area. *Culicoides* diversity found in our trappings is, therefore, not representative for the countries as a whole. In addition, trapping with a single trap type may not accurately represent midge fauna diversity [42, 50, 51]. Nevertheless, because of the consistent study design and use of the same trap type, results can be compared among the three areas in each country and habitats in this study. Midge species and their abundance can be under- or over-estimated compared to the biting rate on livestock animals [52]. Although mostly female biting midges were trapped, these were most likely not host-seeking midges, as they were attracted by a UV-light source [53]. The exact attraction mechanism for female midges towards the trap is thus far unknown. Nonetheless, the OVI trap seems to be the only effective midge trap currently available [54], and this stresses the need for appropriate monitoring methods against biting midges.

A study in Germany showed that adult midges can be trapped during winter months [16]. Although midge numbers captured in our traps were reduced in the months just before and after the winter period, it is not clear whether midges ceased their activity in our study. In addition to their activity during winter, the larval habitats of known midge vectors have not been extensively investigated [55–58]. With more knowledge on the habitats for both *Culicoides* larvae and adults, it will be possible to better understand the factors influencing differences in communities among countries and habitats.

Although the current study revealed differences in biting midge communities among habitats within countries, the underlying factors for this were not identified. Biting midge species diversity was surprisingly high in peri-urban habitats (Table 1), possibly as only few specimens of different species were captured in comparable numbers in this habitat type. In contrast, diversity was lowest at farms in all three countries, which could be explained by the high abundance of the Obsoletus group. Several of the farm collections had high abundance of midges and were therefore sub-sampled for identification. A few individuals of new species may be found if all individuals of farm samples would be identified. However, testing this scenario by simulating additional species in our dataset did not change our conclusions on diversity indices and the dominance of the Obsoletus group in farm habitats. The overwhelming abundance of this species group in farm habitats suppresses the influence on diversity measures of other species occurring on farms. Possibly, specific larval or adult habitats are present on farms that cause species from the Obsoletus group to flourish, while other species may not take advantage of these habitats.

Chaves et al. [59] suggested that lower diversity of vector communities is expected to increase the risk of amplification and spread of a vector-borne disease, because lower vector species diversity is thought to be correlated with higher relative abundance of some species within the community. This is in line with our findings, since the country (Italy) and habitat (farms) with the lowest diversity indices had the highest abundance of midges from the Obsoletus group. On the other hand, a theoretical study by Roche et al. [60] suggested that greater vector species richness leads to higher abundance, and can therefore amplify pathogen transmission. These authors showed that specific vector species in these complex community dynamics could be essential in epidemic take-off, even if these vectors are weakly susceptible to pathogen infection. Given that vector-borne diseases would require a set of multiple species that together influence the rate of transmission, understanding the species composition of vector communities and their interactions with pathogens is becoming increasingly important. Ultimately, entomological field data should be used for the development of mathematical and statistical models, to more accurately assess the effect of environmental factors on midge population dynamics and how this influences disease risks.

## Conclusions

A core European midge community could be identified, with important disease vector species from the Obsoletus group, *C. punctatus* and *C. pulicaris*, as the most abundant in this core community. The presence of a core community throughout Europe suggests that disease dynamics can be homogenous, since the core community is present in all countries and habitats. However, in-depth analysis of the complete midge community composition showed that differences were more distinct among countries than habitat types. In other words, although some midge species are found throughout Europe, distinctive communities can be found within each country. This suggests that specific species within countries may impose a more heterogeneous disease dynamics than expected when only looking at the core community. Although we have elucidated how species communities differ among countries and habitats, it is still unclear how these specific species and their associated communities affect disease risk.

## Additional files

Additional file 1: **Table S1.** A list of the species per country found in this study. References to previous research of *Culicoides* fauna in the same countries are made, as well as remarks on the distribution of the species as described in the IIKC. (XLSX 14 kb)

## Abbreviations

AHSV: African horse sickness virus
BTV: Bluetongue virus
CORINE: Coordination of information on the environment
IIKC: Interactive Identification Key for *Culicoides*
NMDS: Non-metric multidimensional scaling
OVI: Onderstepoort Veterinary Institute
SBV: Schmallenberg virus
